# Evaluation of a phenotype imputation approach using GAW20 simulated data

**DOI:** 10.1186/s12919-018-0134-9

**Published:** 2018-09-17

**Authors:** Yuning Chen, Gina M. Peloso, Josée Dupuis

**Affiliations:** Department of Biostatistics, Boston University School of Public Health, 801 Massachusetts Ave 3rd Floor, Boston, MA 02118 USA

## Abstract

Statistical power, which is the probability of correctly rejecting a false null hypothesis, is a limitation of genome-wide association studies (GWAS). Sample size is a major component of statistical power that can be easily affected by missingness in phenotypic data and restrain the ability to detect associated single-nucleotide polymorphisms (SNPs) with small effect sizes. Although some phenotypes are hard to collect because of cost and loss to follow-up, correlated phenotypes that are easily collected can be leveraged for association analysis. In this paper, we evaluate a phenotype imputation method that incorporates family structure and correlation between multiple phenotypes using GAW20 simulated data. The distribution of missing values is derived using information contained in the missing sample’s relatives and additional correlated phenotypes. We show that this imputation method can improve power in the association analysis compared with excluding observations with missing data, while achieving the correct Type I error rate.

We also examine factors that may affect the imputation accuracy.

## Background

Genome-wide association studies (GWAS) have uncovered thousands of single-nucleotide polymorphisms (SNPs) associated with complex traits [1]. The power of GWAS is limited by the number of individuals with data available for the trait of interest. For easily phenotyped traits, tens of thousands of individuals are typically contributing to GWAS. But the lack of statistical power can still occur from missingness in phenotypic data. Some phenotypes are difficult to collect because of cost, loss to follow-up, and inaccessibility of the biological sample at the time of the study. However, data collected on related phenotypes or from the missing sample’s relatives can be exploited. Current approaches to handle missing data include multiple imputation [2], intermediate phenotype analysis [3], and a recently published method, PhenIMP [4].

In this paper, we first describe an extension to the PhenIMP method, which is designed for performing GWAS on a phenotype with high missingness. Because this imputation approach uses both family structure and additional correlated phenotypes, it has higher power than methods using phenotypic data alone. We make several modifications to this method when applying the approach to the GAW20 simulated data. First, we directly estimate the correlation between multiple phenotypes using the maximum likelihood estimator (MLE) rather than collecting a pilot data set for independent estimation. Second, we combine the observed and imputed individual level data to assess association between genotypes and phenotypes instead of using a fixed effect meta-analysis to combine the analysis performed separately on the observed and imputed data set. We use the GAW20 simulated data to evaluate this method and show that a higher power can be achieved using the imputed data set compared with incomplete data and control the Type I error rate within an acceptable range. We also examine imputation accuracy by varying the missing percentage and under different phenotype correlations. To evaluate power and Type I error rate, we perform our analysis with knowledge of the “answers.”

## Methods

We first introduce the approach to impute the missing phenotypes using a multivariate normal (MVN) statistical framework. Then we describe how we use the simulated data set to compare statistical power and Type I error rate.

### Extension to PhenIMP method

We only consider the scenario where 2 phenotypes are collected, as this is how the pre- and post-treatment triglyceride (TG) level data provided in the GAW20 data set were simulated. This approach can be easily extended to studies with more than 2 phenotypes by changing the dimension of the vectors and matrices.

Let ***Y*** be a vector of length *2n*, where *y*_*1,1*_ *… y*_*1,n*_ are the values of the first phenotype for individual *1* to *n,* and *y*_*2,1*_ *… y*_*2,n*_ are the values for the second phenotype. Missingness can occur in both phenotypes. We assume that the phenotype vector ***Y*** follows a multivariate normal distribution. The expectation vector of ***Y*** is $\boldsymbol{\mu} =\left[{}_{{\boldsymbol{\mu}}_{\mathbf{2}}}^{{\boldsymbol{\mu}}_{\mathbf{1}}}\right],$ where ***μ***_***k***_ is the vector with each element as the observed mean of phenotype *k*. One can divide the unconditional covariance of ***Y*** into the polygenic variance component and the environmental variance component as

1 $$ \sum ={\sum}_A\otimes \upphi +{\sum}_E\otimes I $$

where ⊗ represents the Kronecker product of 2 matrices and

2 $$ {\sum}_A=\left(\begin{array}{cc}{\sigma}_{A_{11}}^2& {\sigma}_{A_{12}}\\ {}{\sigma}_{A_{21}}& {\sigma}_{A_{22}}^2\end{array}\right),{\sum}_E=\left(\begin{array}{cc}{\sigma}_{E_{11}}^2& {\sigma}_{E_{12}}\\ {}{\sigma}_{E_{21}}& {\sigma}_{E_{22}}^2\end{array}\right) $$

Let ${\sigma}_{A_{kk}}^2$ and ${\sigma}_{E_{kk}}^2$ indicate the polygenic and environmental variance of phenotype *k*, respectively, and ${\sigma}_{A_{k1}}$ and ${\sigma}_{E_{k1}}$ indicate the polygenic and environmental covariance between phenotypes *k* and *l,* respectively. All these quantities can be estimated using MLE in the SOLAR [Sequential Oligogenic Linkage Analysis Routines] software [5]. The matrix ɸ describes the correlation between 2 individuals. The kinship matrix can be derived using pedigree structure in family studies or empirically estimated using genotypes. The distribution of ***Y*** can be represented compactly using a block matrix

3 $$ \left(\begin{array}{c}{\boldsymbol{Y}}_{\boldsymbol{m}}\\ {}{\boldsymbol{Y}}_{\boldsymbol{o}}\end{array}\right)\sim MVN\left(\left(\begin{array}{c}{\boldsymbol{\mu}}_{\boldsymbol{m}}\\ {}{\boldsymbol{\mu}}_{\boldsymbol{o}}\end{array}\right),\left[\begin{array}{cc}{\sum}_{mm}& {\sum}_{mo}\\ {}{\sum}_{om}& {\sum}_{oo}\end{array}\right]\right) $$

where, ***Y***_***m***_ is the vector of the missing values in the phenotype vector ***Y*** (missing data) and ***Y***_***o***_ is the vector of all remaining elements (observed data). The parameters ***μ***_***m***_ and are the corresponding vectors of the expectation ***μ***. ∑_*mm*_, ∑_*mo*_, ∑_*om*_ and ∑_*oo*_ are the corresponding block matrices of ∑.

The conditional distribution of ***Ym*** ∣ ***Yo*** follows a multivariate normal distribution, where the mean is computed as

4 $$ \boldsymbol{E}\left({\boldsymbol{Y}}_{\boldsymbol{m}}|{\boldsymbol{Y}}_{\boldsymbol{o}}\right)={\boldsymbol{\mu}}_{\boldsymbol{m}}+{\sum}_{mo}{\sum}_{oo}^{-1}\left({\boldsymbol{Y}}_{\boldsymbol{o}}-{\boldsymbol{\mu}}_{\boldsymbol{o}}\right) $$

We use the estimated ***E***(***Y***_***m***_| ***Y***_***o***_) as the imputed values for the missing data in both phenotypes. The second term in Eq. (4) shows that the imputed values depend on the family structure of the missing samples, expressed by ${\sum}_{mo}{\sum}_{oo}^{-1}$, and also the observed phenotypes of the missing samples and their family members by ***Y***_***o***_ ***− μ***_***o***_. We then run the association analysis on the combined observed and imputed phenotype values.

### Type I error rate evaluation

To select SNPs unassociated with the log-transformed average difference in TG levels, we only include chromosomes 21 and 22 in the analyses as there are no causal and background polygenic SNPs on these 2 chromosomes. The analysis includes 19,763 SNPs. We investigate the Type I error rate using 2 types of outcomes: average difference and single difference measurement. The average difference outcome is defined as the difference between the pre-treatment (average of visits 1 and 2) and post-treatment (average of visits 3 and 4) of the log-transformed TG levels, whereas the single difference measurement outcome is defined as the difference between visit 1 and visit 3 of the log-transformed TG levels. We run the association analyses on 2 data sets to compare Type I error rates: incomplete (omitting observations with missing data) and imputed. We create the incomplete data set by removing all samples with at least one missing visit 3 and/or visit 4 TG value. In the imputed data set, all missing visit 3 TG values are imputed using the method described above. Given the very small missing percentage in visits 1 (4 samples) and 2 (1 sample) and that there are no samples with visits 1 and 2 missing, we fill in the missing visit 1 or visit 2 values using the observed visit 2 or visit 1 values, respectively.

A linear mixed-effects model [6] is performed with log-transformed difference in TG levels (or average difference) as the dependent variable, SNP as the independent variable, and a random effect to account for familial correlation estimated from the pedigree structures. The analyses are repeated 200 times using the 200 GAW20 simulated data sets.

### Power evaluation

We use the 5 major causal SNPs described in the GAW20 Simulation Solutions (rs9661059, rs736004, rs1012116, rs10828412, and rs4399565) to evaluate power in association analyses. The proportion of the trait variance explained by the 5 SNPs is set to 0.125, 0.10, 0.075, 0.05, and 0.025, respectively, in the GAW20 simulation models. As described for the Type I error rate evaluation, a linear mixed-effects model is performed on the 5 causal SNPs in each of 200 iterations. Power is evaluated using both average difference and single difference outcome in the incomplete and imputed data set. All statistical analyses are implemented using the R package *seqMeta* [7].

### Imputation accuracy evaluation

We compute the mean square error (MSE) between the true and the imputed phenotype values to assess imputation accuracy. We first remove 4 samples with missing visit 1 TG value and 1 sample with missing visit 2 TG value to make a data set that has no missing values on visits 1, 2, and 4. To examine imputation accuracy under different missing percentages, we randomly select 20, 50%, or 80% of visit 2 TG values to be missing. To evaluate the effect of phenotypes correlation on the imputation accuracy, we impute the missing visit 2 TG values using visit 1 TG values (correlation = 0.9) and visit 4 TG values (correlation = 0.8), respectively. MSE between the true and the imputed visit 2 TG values is then computed in all 6 combinations of missing percentage and correlation. We repeat 200 times using the 200 simulated data sets.

## Results

There are 680 samples with both genotype and phenotype data available. When evaluating Type I error rate and power, the sample size of the incomplete and imputed data set is 563 and 680, respectively, and it is 675 when evaluating imputation accuracy.

To examine Type I error rate, we use 19,763 SNPs on chromosomes 21 and 22 in each iteration and repeat 200 times for a total of 3,952,600 unassociated SNPs. Type I error rate is computed under 3 different significance levels: 0.05, 1 × 10^− 3^ and 1 × 10^− 4^. A slightly inflated Type I error rate is seen in the incomplete data set when using the average difference outcome.

However, the Type I error rate can be controlled within an acceptable range in the imputed data set for both average difference and single difference measurement outcomes; Table 1 lists the Type I error rate results.

**Table 1:** Simulation result of Type I error rate

	Average difference		Single difference	
	Incomplete	Imputed	Incomplete	Imputed
α = 0.05	0.046	0.046	0.046	0.046
α = 1 × 10^−3^	1.01 × 10^− 3^	8.79 × 10^−4^	8.29 × 10^−5^	8.4 × 10^− 4^
α = 1 × 10^− 4^	1.25 × 10^− 4^	9.22 × 10^− 5^	8.67 × 10^− 5^	8.18 × 10^− 5^

To evaluate power, we restrict our attention to the 5 known major causal SNPs. We compute power under 2 different significance levels: 0.01, accounting for the 5 SNPs tested, and 1 × 10^− 4^, a more stringent threshold. Analyses using average difference as the outcome outperform analyses using single difference measurement for all 5 SNPs (Tables 2 and 3). For example, in imputed data, rs9661059 has 89% power to detect a true association with the average difference as the outcome compared with 56.5% power with a single difference measurement at an alpha of 0.01. We observed higher power in the imputed data set than in the incomplete data set. For example, rs736004 has 27 and 39% power to detect the true association with average difference as the outcome at an alpha of 1 × 10^− 4^ using the incomplete and imputed data, respectively. A similar pattern can be found when detecting the association using single difference outcome, where rs736004 has 16 and 32.5% power using the incomplete and imputed data, respectively.

**Table 2:** Simulation result of power when *P* < 0.01

	Average difference		Single difference	
	Incomplete	Imputed	Incomplete	Imputed
*rs9661059*	0.745	0.890	0.475	0.565
*rs736004*	0.885	0.955	0.875	0.960
*rs1012116*	0.660	0.705	0.480	0.650
*rs10828412*	0.875	0.965	0.390	0.755
*rs4399565*	0.105	0.250	0.010	0.035

**Table 3:** Simulation result of power when *P* < 1 × 10–4

	Average difference		Single difference	
	Incomplete	Imputed	Incomplete	Imputed
*rs9661059*	0.175	0.260	0.020	0.040
*rs736004*	0.270	0.390	0.160	0.325
*rs1012116*	0.120	0.120	0.025	0.070
*rs10828412*	0.200	0.490	0.010	0.060
*rs4399565*	0.000	0.045	0.000	0.000

When assessing imputation accuracy, the MSE is relatively stable among different missing percentages (Table 4). The average MSEs of 20, 50, and 80% missing percentages are 0.0653, 0.0661, and 0.0693 when imputing from visit 1 TG values, and it goes from 0.1172 to 0.1235 when imputing from visit 4 TG values.

**Table 4:** Simulation result of imputation accuracy

Missing percentage	MSE	
	*p* = 0.9	*p* = 0.8
20%	0.0653	0.1172
50%	0.0661	0.1190
80%	0.0693	0.1235

## Discussion and conclusions

We described a phenotype imputation method incorporating family structure and additional phenotypes, and evaluated the performance of this method using GAW20 simulated data. This method can be applied to unrelated or family data by simply changing the correlation matrix ɸ. We show that the imputed missing phenotypes can be determined under a MVN distribution using family structure, an observed second phenotype of the missing samples, and the 2 observed phenotypes of the missing sample’s family members.

We found that the average difference outcome performed better than the single difference measurement outcome. The association analysis for the average difference outcome is more accurate than the single difference measurement outcome because the averaging process decreases the variance in average difference outcome. The imputed data set provided higher power than the incomplete data set, the latter being the most common way to handle missing data in GWAS, and there was no inflated Type I error in the imputed data set. The adequate control of Type I error with the modifications to the PhenIMP method suggests that the MLE estimation for the phenotypes correlation and joint analyses of the observed and imputed individual level data can be performed. Even though the imputation accuracy is relatively stable among different missing percentages, a higher correlation between phenotypes can improve the imputation accuracy. Other factors, such as effect size, need further investigation.
